# Orthogonal mode couplers for plasmonic chip based on metal–insulator–metal waveguide for temperature sensing application

**DOI:** 10.1038/s41598-024-54244-0

**Published:** 2024-02-12

**Authors:** Muhammad Ali Butt, Ryszard Piramidowicz

**Affiliations:** Institute of Microelectronics and Optoelectronics, Warsaw University of Technology, Koszykowa 75, 00-662 Warsaw, Poland

**Keywords:** Metal–insulator–metal waveguide, Surface plasmons, Orthogonal couplers, Momentum mismatch, Temperature sensing, Polydimethylsiloxane, Plasmonics, Applied optics, Optical materials and structures, Other photonics

## Abstract

In this work, a plasmonic sensor based on metal–insulator–metal (MIM) waveguide for temperature sensing application is numerically investigated via finite element method (FEM). The resonant cavity filled with PDMS polymer is side-coupled to the MIM bus waveguide. The sensitivity of the proposed device is ~ − 0.44 nm/°C which can be further enhanced to − 0.63 nm/°C by embedding a period array of metallic nanoblocks in the center of the cavity. We comprehend the existence of numerous highly attractive and sensitive plasmonic sensor designs, yet a notable gap exists in the exploration of light coupling mechanisms to these nanoscale waveguides. Consequently, we introduced an attractive approach: orthogonal mode couplers designed for plasmonic chips, which leverage MIM waveguide-based sensors. The optimized transmission of the hybrid system including silicon couplers and MIM waveguide is in the range of − 1.73 dB to − 2.93 dB for a broad wavelength range of 1450–1650 nm. The skillful integration of these couplers not only distinguishes our plasmonic sensor but also positions it as a highly promising solution for an extensive array of sensing applications.

## Introduction

Plasmonics, a subdivision of nanophotonics, carries significant importance in contemporary science and technology^1^. This domain delves into the interactions between electromagnetic (EM) waves and unbound electrons within tiny metallic structures, leading to the creation of surface plasmons (SPs) that can confine and control light at the nanoscale. The impact of plasmonics extends widely, with practical applications in diverse fields like optics^2^, sensing^3,4^, and information technology^5^. Plasmonic structures can be designed to amplify the sensitivity and precision of sensors, enabling the detection of exceedingly small quantities of molecules or nanoparticles^6,7^. In the optical realm, plasmonics have triggered a revolution in the development of ultra-compact photonic devices, presenting the potential for more rapid, compact, and energy-efficient optical components. Additionally, plasmonics play a key role in advancing data storage and information processing, promising quicker data transfer rates and the emergence of pioneering technologies such as plasmonic circuits^5,8^. Ultimately, plasmonics has emerged as a transformative discipline with profound implications for the future of science and technology.

A metal–insulator–metal (MIM) structure is one of the plasmonic waveguides which serves as a fundamental element within the realm of photonics and plasmonics, with the primary purpose of directing and controlling EM waves operating at optical frequencies^9^. This structure consists of two parallel metal layers separated by a dielectric insulator (air, n = 1.0), enabling the confinement and transmission of surface plasmon polaritons (SPPs), which are collective electron oscillations occurring at the interfaces between the metal and dielectric^10^. MIM waveguides exhibit remarkable efficiency in both confining and guiding light at scales smaller than the wavelength of light, rendering them exceptionally suitable for various applications in nanophotonics, sensing, and signal processing^11^. Their capacity to confine light into minute volumes and their potential for tailored adjustments in their geometrical attributes make MIM waveguides a versatile instrument for harnessing the characteristics of plasmonic waves across a spectrum of photonic devices^7,12,13^.

Coupling light to a MIM waveguide presents several challenges linked to the design and fabrication of these waveguides^14^. Notable among these issues are mode matching, as the incident light must be precisely aligned with the waveguide's mode for efficient coupling^15^. Achieving optimal mode matching becomes particularly complex when dealing with diverse light sources and waveguide structures. Another significant concern is the high optical losses associated with MIM waveguides, stemming from metal absorption and scattering. These losses can severely affect the efficiency of light coupling, prompting the need for strategies to reduce them. Additionally, the fabrication tolerances of MIM waveguides play a crucial role in their performance. Variations in dimensions and geometries due to fabrication processes can make it challenging to achieve consistent and efficient coupling. Moreover, MIM waveguides often exhibit strong polarization dependence, meaning the efficiency of light coupling may vary with the incident light's polarization orientation, necessitating polarization control and management techniques. Addressing these problems is essential to harness the full potential of MIM waveguides in various photonic applications.

In this work, we introduced a simple yet highly effective design for a plasmonic sensor, specifically tailored for temperature sensing applications. This sensor capitalizes on the remarkable properties of a MIM waveguide, which forms the core of our device. At the heart of our plasmonic sensor lies a resonant cavity, meticulously engineered to be filled with a responsive thermo-optic polydimethylsiloxane (PDMS) polymer. This polymer material demonstrates a highly sensitive response to variations in ambient temperature, making it the ideal candidate for our sensing application. Furthermore, we have implemented orthogonal couplers at both the input and output interfaces of the MIM waveguide to couple the light in and out of the plasmonic waveguide. This smart integration of the couplers further sets our plasmonic sensor apart, making it a promising solution for a wide range of temperature-sensing applications.

## Results

In our previous works on MIM waveguide-based plasmonic sensors, we suggested Si^16^ and Si_3_N_4_^17^ tapered junctions Butt-coupled to the MIM waveguide for efficient mode conversion. However, it requires precise fabrication techniques and waveguide alignment for efficient mode coupling. Motivated by the work of Lau et al. which introduced an innovative and highly compact coupling scheme that is different from the conventional approaches^18^. This conceptually unique orthogonal coupling scheme involves positioning the dielectric waveguide and the MIM plasmonic waveguide in a perpendicular orientation. Notably, this configuration showcases impressive efficiency in the coupling of light.Even though the dielectric and MIM waveguides have vastly distinct lateral dimensions, with a dielectric/plasmonic waveguide width aspect ratio of approximately 0.1, Lau et al. calculated notable coupling efficiencies of up to 70% in a 3D context, assuming the use of silver as the metal^18^. To validate these promising calculations, experimental results support the notion that efficient coupling, at approximately 50%, is consistently maintained across a wide bandwidth. Recently, Tanyi et al. proposed a design of a plasmonic modulator based on hybrid orthogonal silver junctions using vanadium dioxide as the modulating material on the SOI platform^19^. These findings underscore the viability of the orthogonal coupling scheme as a powerful tool for achieving high-efficiency light coupling in compact, plasmonic-based systems.

SPP modes have a momentum mismatch with plane waves or conventional dielectric modes, and standard techniques like prisms or Bragg gratings are used to address this issue^20^. However, when dealing with plasmonic MIM waveguide modes, which consist of two closely coupled SPP surface modes, these standard coupling techniques become less practical due to the extremely confined nature of the mode, making it difficult to achieve efficient coupling to the MIM waveguide^21,22^. The limited coupling between the silicon waveguide and the MIM waveguide primarily arises from a substantial disparity in the momentum of their fundamental modes. This momentum mismatch can be attributed to two primary factors^23^. The first factor is rooted in the inherent difference in momentum between the SPP mode on a single interface and conventional waveguide or plane waves, necessitating specialized coupling techniques as previously discussed. The second factor results from the significantly smaller dimensions of the slot in the MIM waveguide, typically being about ten times smaller than those of the conventional silicon waveguide.

The orthogonal coupling scheme presented in this work offers a promising solution to the momentum mismatch issue without the need for additional tapering. This innovative approach focuses on aligning the orthogonal momentum component of the silicon waveguide (*k*_*x*_), with the momentum component of the MIM waveguide, rather than the conventional approach (prism or Bragg grating), which targets the longitudinal component. Notably, the *k*_*x*_ component exhibits a relatively small mismatch with the *k*_*SPP*_ over a broad bandwidth. To effectively channel power into the MIM waveguide, it is essential to spatially synchronize the *k*_*x*_ component with the *k*_*SPP*_ component. This strategic alignment enables efficient coupling of optical energy to the plasmonic slot waveguide, circumventing the need for additional tapering techniques^18^.

**Figure 1:**
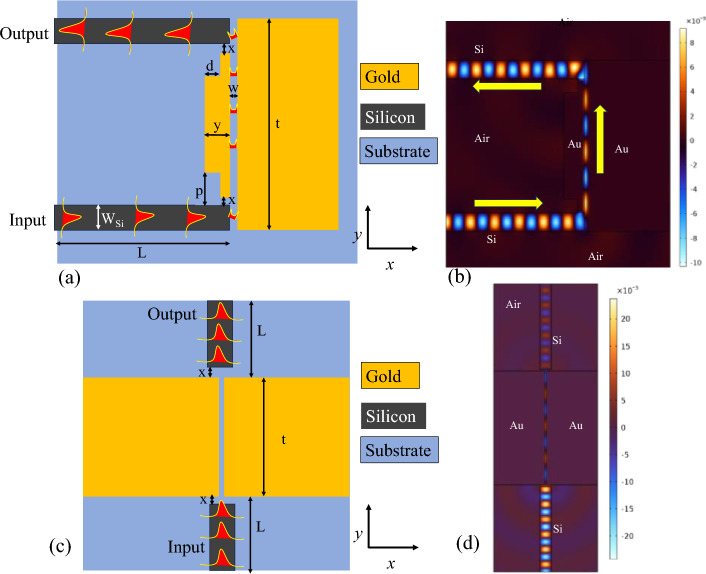
Schematic representation of (**a**) orthogonal coupler, (**c**) Butt coupler. Normalized H-field distribution at the operational wavelength of 1550 nm in (**b**) orthogonal coupling arrangement, (**d**) Butt coupling arrangement.

Within our device design, we have harnessed the exceptional properties of gold (Au) and air to assemble the critical components of the MIM waveguide. Au, revered for its plasmonic characteristics, is employed as a metal, while air serves as the dielectric material. The Lorentz-Drude model $\left( {\varepsilon = \varepsilon_{\infty } - \frac{{\omega_{p}^{2} }}{{\omega^{2} + j\omega \gamma }}} \right)$ is used to compute the permittivity of Au^16^. Where $\varepsilon_{\infty } = 9.0685$, $\omega_{p} = 135.44 \times 10^{14} \;{\text{rad/s}}$, and $\gamma = 1.15 \times 10^{14} \;{\text{ rad/s}}$. For the sake of simplicity, we have set the width (w) of a MIM waveguide to a constant value of 50 nm. This choice is informed by the fact that a 50 nm width is capable of supporting a fundamental TM_0_ mode, and furthermore, it can be precisely patterned using E-beam lithography, allowing for an exceptional resolution of less than 10 nm^24^. This strategic choice of materials underpins the high-performance capabilities of our waveguide. To facilitate seamless light coupling, we have resourcefully integrated orthogonal couplers constructed from silicon ridge waveguides. These waveguides are designed with a fixed width of 400 nm, ensuring robust support for the fundamental mode within the wavelength range of 1450–1650 nm. This range covers a significant portion of the optical spectrum, making our design versatile and adaptable to various applications. The optical constants of silicon material are taken from^25^. For a more comprehensive understanding of our design, we offer a visual representation in Fig. 1. In Fig. 1a, a schematic depiction of the embedded orthogonal couplers within the MIM waveguide can be seen, highlighting their strategic placement. The MIM waveguide is designed with precision, featuring a nanometer-scale slot or gap sandwiched between two metal claddings on opposing sides. The geometric configuration of metal cladding which is between the silicon input and output waveguides defined by parameters such as x, y, d, and p, plays a pivotal role demanding meticulous optimization to mitigate transmission loss. Simultaneously, the complementary metal cladding segment adopts a standardized form, adeptly designed to foster the confinement and seamless propagation of SPP waves along the metal–dielectric interface. Meanwhile, Fig. 1b presents a visualization of the H-field distribution at the fundamental wavelength of 1550 nm.

In our endeavor to evaluate the effectiveness of orthogonal couplers, we have taken an additional step by simulating an alternative arrangement of waveguides known as Butt-coupling. Butt-coupling of light is a technique where optical components are directly aligned with minimal spacing between them^26^. This method allows for efficient light transmission but has certain limitations. It is highly sensitive to precise alignment, making even minor misalignments detrimental to optical power and efficiency. Surface imperfections and thermal effects can also negatively impact performance. As such, butt-coupling is a method that requires careful handling and consideration of these factors for optimal results in optical systems.

As illustrated in Fig. 1c, this configuration offers a valuable point of comparison, enabling us to evaluate the relative advantages of our chosen design. The particulars of these simulations are further illuminated in Fig. 1d, where we present the normalized H-field distribution at the critical wavelength of 1550 nm. This vivid visualization allows for a detailed examination of the electromagnetic field behavior, providing insights into the performance of our couplers. To ensure a truly equitable evaluation, we have maintained consistent dimensions for both configurations (Table 1). This persistent approach ensures that any differences in performance can be attributed solely to the coupling arrangements, enabling us to draw meaningful conclusions about the superiority of our orthogonal couplers.

**Table 1:** The geometric parameters of the orthogonal coupler utilized in this study.

Variables	Description	Values (nm)
W_Si_	Width of silicon waveguide	400 (fixed)
L	Length of silicon waveguide	3000 (fixed)
w	Width of MIM waveguide	50 (fixed)
p	Separation between silicon waveguide and metal layer to avoid losses	300 (fixed)
t	Length of MIM waveguide	2000–4500
y	Width of Au layer on the side of couplers	450 (fixed)
d	Depth of the etched part of Au	10–450
x	Gap between silicon waveguide and metal layer	0–60
Footprint	Total size of the chip (lengthxwidth)	6 × 6 µm^2^ (Orthogonal coupler) 10 × 6 µm^2^ (Butt coupler)

In the realm of ideal conditions, our assumptions guide us to specific parameters: w = 50 nm, y = 450 nm, p = 450 nm, and x = 0 nm. However, the critical variable in this context is d, which demands careful optimization to attain the ideal width of the Au layer (denoted as y–d) which is in direct contact with the silicon waveguide, where the ultimate transmission efficiency is achieved. When a substantial metal layer, such as Au or Ag, intimately interfaces with a silicon waveguide, it initiates a cascade of significant consequences, often deleterious to the optical device's performance. One of the most notable impacts is the initiation of compelling plasmonic losses. Plasmons, those collective oscillations of free electrons within the metal, result in the dissipation of energy from the guided modes in the waveguide. This, in turn, leads to increased optical losses, thereby diminishing the overall efficiency of the waveguide and the entire optical device. It's important to emphasize the high absorptivity of metals in the optical spectrum, signifying their capacity to capture a substantial portion of incident light energy. Furthermore, metal surfaces tend to scatter light, causing the diversion of optical power away from the intended guided mode. This dual action of absorption and scattering collectively yields reduced light transmission through the waveguide, presenting a difficult challenge to the device's functionality.

**Figure 2:**
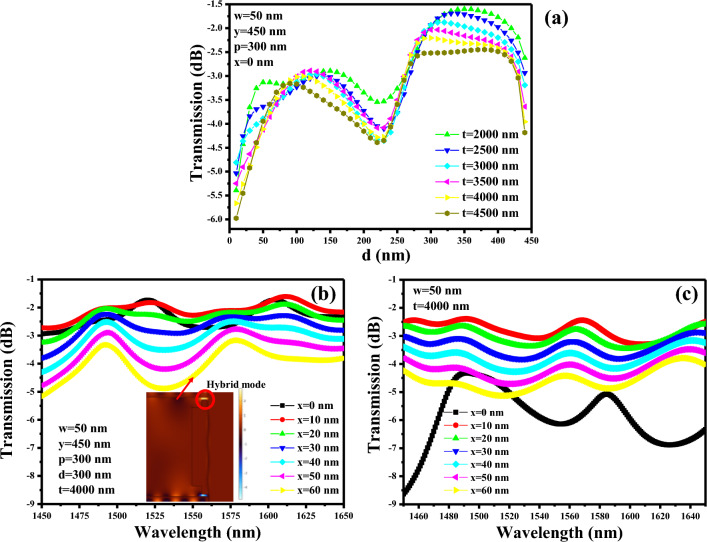
Optimization of the coupler segment, (**a**) transmission of orthogonal coupler versus d, (**b**) transmission of orthogonal coupler versus x over a wavelength range 1450–1650 nm. Inset shows the energy confinement between Au and metal forming a hybrid mode, (**c**) transmission of Butt-coupler versus x over a wavelength range of 1450–1650 nm.

Moreover, when an Au layer directly engages with the silicon waveguide, it significantly alters the mode properties of the waveguide itself. This includes the transformation of the mode profile, effective refractive index, and propagation characteristics. Such alterations invariably induce shifts in mode dispersion and confinement, thereby impacting the essential functionality of the device. The measure of our device’s effectiveness is encapsulated in the transmission, calculated as $T \left( {dB} \right) = \frac{{P_{out} }}{{P_{in} }}$. Here, P_out_ and P_in_ correspond to the output and input powers, respectively, noted at the output silicon waveguide and input silicon waveguide. This comprehensive calculation encompasses multiple factors, including the propagation loss of the MIM waveguide, the propagation loss of the silicon waveguide at both input and output, as well as the mode conversion losses (from dielectric to plasmonic and vice versa). Careful consideration of these parameters is essential for accurately assessing the performance of our optical device. The transmission value oscillates between − 1.59 and − 5.97 dB when d is varied between 0 and 450 nm, which also slightly depends on t (length of MIM waveguide). We must carefully select the length of the MIM waveguide to ensure a well-defined separation between the resonant cavity and the silicon waveguides (both input and output). Consequently, we maintain t at 4000 nm, a value conducive to accommodating both the input and output silicon waveguides, along with the cavity (R = 480–500 nm) in our sensor design. This meticulous design choice allows us to achieve a transmission of − 2.2 dB at d = 300 nm, as depicted in Fig. 2a.

In the next phase of our investigation, we aim to assess the impact of varying the parameter x on the transmission characteristics of the coupler across a wide wavelength spectrum. The remaining geometric parameters, namely w, y, p, d, and t, are held constant at 50 nm, 450 nm, 300 nm, 300 nm, and 4000 nm, respectively. When x is set to 0 nm, the coupler’s transmission falls within the range of − 1.73 dB to − 2.93 dB for wavelengths spanning from 1450 to 1650 nm. This then transitions to a range of − 3.18 dB to − 5.18 dB when x is increased to 60 nm, as illustrated in Fig. 2b. The decline in output power can be attributed to the emergence of a hybrid mode between the silicon and Au layers, as visually depicted in the inset of Fig. 2b. Upon careful examination of Fig. 2b, it becomes evident that any value of x within the range of 0–20 nm is applicable as the gap between the silicon waveguide and the metal layer. However, it is advisable to minimize or eliminate any gap between the silicon waveguide and the Au layer at the coupler junction to maintain optimal transmission performance^18^.

Furthermore, we conducted a comparative analysis between the transmission characteristics of an orthogonal coupler and the Butt-coupling configuration as shown in Fig. 2c. The geometric parameters employed for the Butt-coupling setup closely resemble those used for the orthogonal coupler. In this investigation, the input and output waveguides have been intentionally displaced from the MIM waveguide, enabling us to assess the impact of the parameter ‘x’ on the transmission. In stark contrast to the orthogonal coupler, which attains its optimal transmission performance at x = 0 nm, the transmission for the Butt-coupling configuration exhibits a range of − 4.3 dB to − 8.6 dB at this point. However, even a slight deviation in the value of ‘x’ results in a significant fluctuation in transmission, ranging from − 2.44 to − 5.13 dB for values of x greater than or equal to 10 nm. It is worth noting that Butt-coupling introduces a resonance effect, which has the effect of limiting the operational bandwidth of the system.

## Discussion

**Figure 3:**
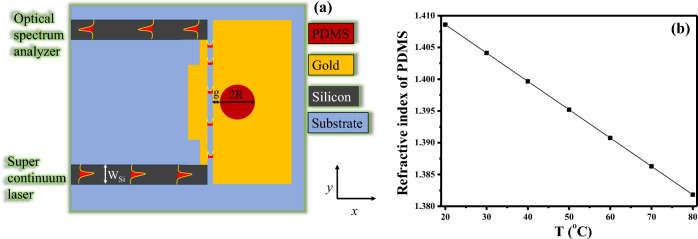
(**a**) Schematic of a plasmonic chip for temperature sensing, (**b**) Refractive index of PDMS versus ambient temperature.

After achieving optimization of the orthogonal couplers to ensure efficient mode conversion, the next step involves fine-tuning the geometric parameters of the sensor design. This design features a circular cavity filled with a PDMS layer, which is side-coupled with the MIM bus waveguide, as illustrated in Fig. 3a. PDMS possesses an intriguing property for temperature sensing due to its responsiveness to changes in temperature^27^. When employed in temperature sensors, PDMS proves to be a highly sensitive material to track and identify temperature fluctuations. In the past few years, several plasmonic sensors utilized PDMS for temperature sensing applications^28–31^.

The refractive index of PDMS exhibits variations in response to temperature changes due to the inherent thermal expansion properties of the material. PDMS is a silicone-based elastomer with a relatively low glass transition temperature. When subjected to an increase in temperature, the polymer chains within PDMS tend to expand and exhibit increased mobility. This expansion leads to a reduction in the material's density, and consequently, the refractive index decreases. Conversely, when PDMS is cooled, the polymer chains contract, resulting in a higher density and a higher refractive index^32^. The changes in the refractive index of the PDMS layer in response to temperature fluctuations can be precisely articulated as follows^31^:

$$ {\text{Refractive index of PDMS}} = 1.4176 - 4.5 \times 10^{ - 4} .{\text{T}} $$

where T is the ambient temperature. The refractive index versus temperature graph is shown in Fig. 3b. The parameter representing the gap between the cavity and the waveguide is denoted as g, while the radius of the cavity is represented by R, varying within the range of 480–500 nm.

The design of plasmonic sensors follows a straightforward approach, primarily hinging on the optimization of two key parameters: R and g. Fine-tuning these parameters is crucial to achieving an optimized resonance dip within the desired spectral range, making the sensor’s design both precise and effective. The optimized geometric parameters of the orthogonal coupler are thoughtfully detailed in Table 2 which are used in this analysis.

**Table 2:** Geometric parameters of the sensor design used in this study.

Variables	Values (nm)	Description
W_Si_	400	Fixed
L	3000	Fixed
w	50	Fixed
t	4000	Optimized
y	450	Fixed
x	0	Optimized
d	300	Optimized
p	300	Fixed
R	480–500	Radius of the circular cavity
g	10, 15, 20, 25, 30, 35	Gap between bus waveguide and cavity

**Figure 4:**
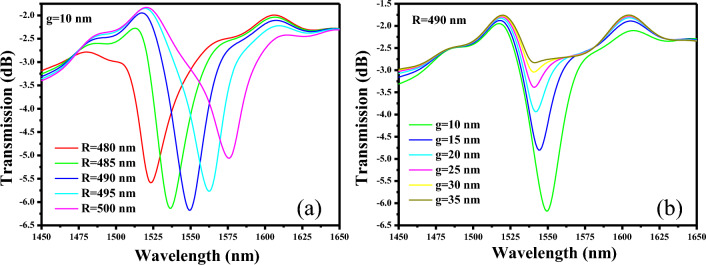
Sensor optimization process, (**a**) transmission versus R, (**b**) transmission versus g. Note that optimized geometric parameters of the orthogonal couplers are used to launch the light in and take it out from the MIM waveguide.

In the initial phase of our investigation, we maintain g at a constant value of 10 nm, while we systematically vary the parameter R within a range spanning from 480 to 500 nm. The objective is to pinpoint a resonance dip within our desired optical spectrum, as classily illustrated in Fig. 4a. As R undergoes this incremental adjustment, an intriguing phenomenon unfolds: the resonance wavelength gradually experiences a redshift, moving from 480 to 500 nm. This redshift manifests as a pronounced resonance dip, yielding specific wavelengths of 1523.2 nm, 1536.5 nm, 1549.4 nm, 1562.2 nm, and 1576.3 nm for R values of 480 nm, 485 nm, 490 nm, 495 nm, and 500 nm, respectively. Furthermore, g is optimized to find the optimum value to assist the maximum transfer of EM wave from the bus waveguide to the cavity. R is fixed at 490 nm which provides a resonance dip at 1549.4 nm, while g is varied between 10 and 35 nm as shown in Fig. 4b. Depth of the resonance dip, i.e., extinction ratio $\left( {ER = 10 \times log\frac{{P_{out} }}{{P_{in} }}} \right)$, where P_out_ and P_in_ are output and input power, respectively, decreases as g increases from 10 to 35 nm. A higher ER stands as a critical indicator of the enhanced ability to distinguish between the on-resonance and off-resonance states, a parameter of utmost significance in photonic sensors. Therefore, we encourage maintaining a fixed gap width (g) of 10 nm, as this choice ensures robust light confinement within the cavity at the resonance wavelength. This strategy ultimately contributes to the achievement of strong resonance conditions and bolsters the sensor's performance.

Temperature sensing is a fundamental aspect of modern science and technology, with a myriad of applications across various industries. These sensors are designed to measure thermal energy and provide crucial data for maintaining safe and efficient operations. In industrial settings, temperature sensors are vital for monitoring and controlling processes in sectors like manufacturing, pharmaceuticals, and energy production^33^. They play a crucial role in climate control systems, ensuring our homes and workplaces are comfortable and energy efficient. Temperature sensors are also essential in healthcare, enabling precise monitoring of a patient's body temperature^34^. In scientific research, they are used to investigate physical and chemical phenomena. Furthermore, environmental monitoring relies on temperature sensors to assess climate change and its impact^35^. From everyday conveniences to critical scientific endeavors, temperature sensing is integral to our modern world.

**Figure 5:**
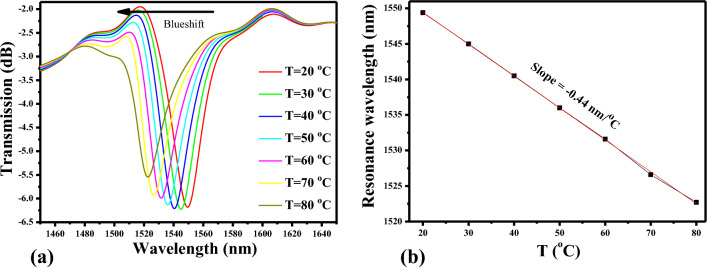
(**a**) Transmission or a plasmonic sensor system versus ambient temperature, (**b**) Dependence of resonance wavelength versus ambient temperature.

The performance of the optimized sensor design was examined across a temperature range spanning from 20 to 80 °C. As previously discussed, it was established that the refractive index of PDMS exhibits a linear trend with temperature. Consequently, as the ambient temperature rises, the refractive index of the PDMS material decreases, leading to a noticeable blueshift in the resonance wavelength, as depicted in Fig. 5a. The sensitivity of a plasmonic sensor is greatly influenced by its type and design. In the realm of plasmonic sensors, the ability to detect changes in ambient temperature is a fundamental and commonly utilized performance characteristic. This sensitivity is typically quantified in terms of bulk RI sensitivity (S), expressed as S (nm/°C) = Δλ/ΔT. Here, Δλ represents the shift in resonance wavelength at which surface plasmons (SP) are excited, and ΔT signifies the change in the ambient temperature. This parameter provides a crucial metric for assessing the sensor's effectiveness in responding to variations in its operating environment.

The resonance wavelength versus ambient temperature is plotted in Fig. 5b. A linear relationship between two sets of variables is established by employing a linear fitting of data. This method seeks to find the best-fit line that minimizes the sum of the squared differences between the observed data points and their corresponding values predicted by the linear model. The equation of the fitted line takes the form of y = mx + b, where ‘y’ represents the dependent variable, ‘x’ is the independent variable, ‘m’ is the slope of the line, and 'b' is the y-intercept. The slope of the line is − 0.44 nm/°C which signifies the sensitivity of the device.

**Figure 6:**
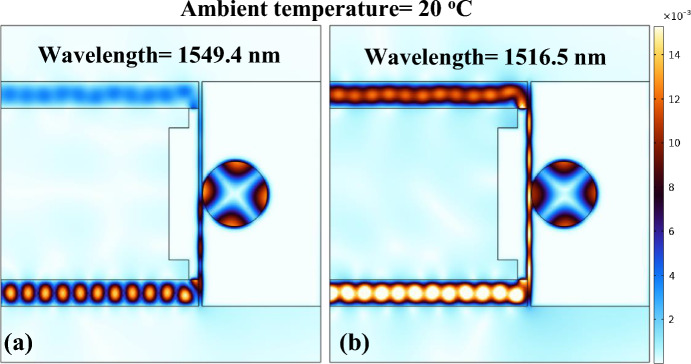
Normalized H-field distribution in the sensor at, (**a**) resonant wavelength = 1549.4 nm, (**b**) non-resonant wavelength = 1516.5 nm.

Figure 6a,b illustrate the normalized H-field distribution within the sensor, specifically at the operational wavelengths of 1549.4 nm and 1516.5 nm, representing the on-resonance and off-resonance states, respectively. Depicted in Fig. 6, the transmission of light is observed in each segment of the sensing device. The light propagating in the silicon input waveguide is then seamlessly channelled into the MIM waveguide, adopting the form of SP wave modes. The light is coupled to the cavity when the resonance condition is satisfied, as illustrated in Fig. 6a. Alternatively, in the off-resonance state, the light continues its propagation within the MIM waveguide, and the SP modes smoothly transform back into the dielectric mode. This transformed light is effectively collected at the silicon waveguide (Fig. 6b).

**Figure 7:**
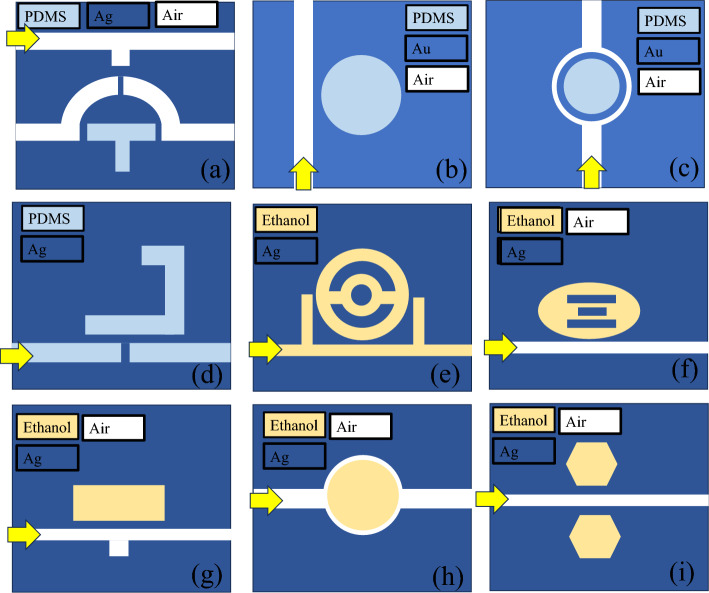
Schematic illustration of temperature sensors based on MIM waveguide cavity filled with either ethanol or PDMS as presented in references^30,36–42^.

Table 3 presents the performance comparison of a few recently proposed plasmonic sensors based on MIM waveguides for temperature sensing applications. The device designs outlined in ^30,36–38^ incorporated PDMS for temperature sensing, whereas^39–42^ utilized ethanol as the sensing material. The choice between ethanol and PDMS for temperature sensing applications depends on specific requirements and the intended use. Ethanol, a commonly used alcohol, has a well-defined and predictable thermal expansion coefficient, making it suitable for temperature sensing in certain environments. It exhibits a relatively linear response to temperature changes, making it straightforward to calibrate. On the other hand, PDMS, a silicone-based polymer, offers flexibility and durability. PDMS can be engineered with embedded sensors for more complex applications and is resistant to various environmental factors. However, its thermal expansion properties may not be as well-defined as those of ethanol. In making the decision, considerations should primarily revolve around factors such as the intended sensitivity, prevailing environmental conditions, and the overarching design specifications essential for the temperature sensing application. These studies employed 2D numerical models to assess the spectral characteristics and the sensing performance of their respective devices. However, a crucial aspect of the sensing system, namely the light coupling mechanism to the MIM waveguide, was not adequately addressed in these works as shown in Fig. 7. In contrast, our research addresses this gap by presenting a comprehensive numerical model of a sensor system. Our sensor design involves the use of PDMS as the sensing material, and the light is efficiently coupled to the MIM waveguide through orthogonal mode couplers.

**Table 3:** Performance comparison of MIM waveguide-based temperature sensors.

Sensor design	Sensing material	Sensitivity (nm/°C)	Simulation model (2D or 3D)	Coupling mechanism	References
Two T-shaped cavities	PDMS	− 0.36	2D	Not present	^36^
Defective oval resonator	Ethanol	− 0.44, − 0.94, − 1.282, − 2.463	2D	Not present	^40^
Concentric double rings resonator	Ethanol	1.48	2D	Not present	^38^
Side-coupled circular cavity Ring encapsulated circular cavity	PDMS	(i) − 0.58, (ii) − 0.64	2D	Not present	^37^
Semi-square ring resonator	PDMS	− 4	2D	Not present	^30^
Dual laterally side-coupled hexagonal cavities	Ethanol	− 0.45	2D	Not present	^39^
Square cavity	Ethanol	− 0.36	2D	Not present	^41^
Ring cavity	Ethanol	− 0.53	2D	Not present	^42^
Circular cavity side coupled to waveguide	PDMS	− 0.44	2D	Yes (orthogonal couplers)	This work
Circular cavity embedded with metallic nanoblocks side coupled to waveguide	PDMS	− 0.63	2D	Yes (orthogonal couplers)	This work

## Methods

### Numerical simulations

We employed COMSOL Multiphysics 6.1 software to conduct simulations of transmittance and field distributions using the 2D-finite element method (2D-FEM). The sub-domains within our device design were meticulously divided into triangular mesh elements, each having a grid size of λ/250. This precise meshing strategy proved essential in ensuring the generation of highly accurate simulation results while efficiently employing our computing resources (8-Core Processor (3.8 GHz); RAM = 128 GB). In the context of analyzing EM wave phenomena, it becomes imperative to establish an open-bounded domain, essentially a computational space where EM waves propagate without encountering reflections. In the pursuit of simulating such an open geometry, scattering boundary conditions (SBC) are employed, which were judiciously applied to the outer borders of the FEM simulation window. This approach allowed us to achieve comprehensive and reliable simulations that closely mimic real-world wave behavior.

### Sensitivity enhancement mechanism

Figure 7 illustrates a compelling trend: sensing devices featuring intricate cavity designs exhibit significantly higher sensitivity compared to their counterparts with simpler and symmetric cavity designs. Consequently, optimizing the sensitivity of the device mandates strategic modifications in the cavity design. Nevertheless, it is imperative to acknowledge that such enhancements may increase the fabrication complexity and precision demands. For instance, the designs presented in^30,36,38,40,41^ utilize Fano resonances for temperature sensing applications which offer high sensitivity. Fano resonance, a phenomenon characterized by an asymmetric spectral profile resulting from the interference between a discrete state and a continuum of states, has proven to be a powerful mechanism for enhancing the sensitivity of plasmonic sensors. In the context of plasmonic sensing, Fano resonance arises when the localized surface plasmon resonance (LSPR) of a nanostructure interferes with a non-resonant background. This intricate interaction leads to a sharp and asymmetric spectral response, making the sensor highly responsive to subtle changes in the surrounding environment.

**Figure 8:**
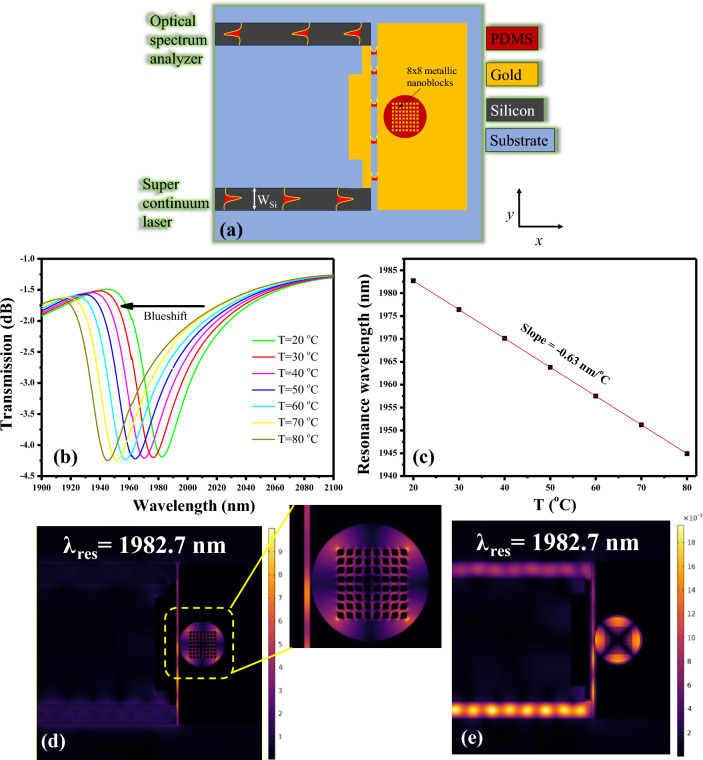
(**a**) Schematic illustration of a plasmonic sensor with cavity embedded with metallic nanoblocks, (**b**) Transmission spectrum of a plasmonic sensor versus ambient temperature, (**c**) Dependence of resonance wavelength versus ambient temperature, (**d**) Normalized E-field distribution in the sensor at resonance wavelength of 1982.7 nm at an ambient temperature of 20 °C, (**e**) Normalized H-field distribution in the sensor at resonance wavelength of 1982.7 nm at an ambient temperature of 20 °C.

To augment the interplay between the SPP wave and the fluctuations in the refractive index of the PDMS layer induced by ambient temperature changes, we strategically incorporated a periodic 8 × 8 array of metallic nanoblocks in the circular cavity as shown in Fig. 8a. The nanoblocks in this system possess a size of 50 nm^2^, meticulously organized in a periodic arrangement with an interval of 25 nm. This strategic placement serves to intensify the system's sensitivity by effectively constraining the E-field within the proximity of the metallic blocks. This confinement optimally facilitates a more refined and responsive reaction to variations in environmental conditions. It’s noteworthy that the remaining geometric parameters, such as the cavity radius (R = 490 nm) and the gap between the cavity and MIM waveguide (g = 10 nm), remain consistent with those employed in generating the transmission plot of a plasmonic sensor, as illustrated in Fig. 5a. This cohesive approach ensures a coherent comparison and analysis of the system's performance in different design setups.

In Fig. 8b, the observable shift of the resonance dip towards the higher wavelength spectrum range prompts us to focus our analysis on the transmission spectrum between 1900 and 2100 nm. Notably, the resonance wavelength undergoes a discernible blueshift with increasing ambient temperature, mirroring the trend observed in Fig. 5a. The correlation between resonance wavelength and ambient temperature is graphically depicted in Fig. 8c, revealing a slope of -0.63 nm/°C. This numerical insight underscores a substantial 43% increase in sensitivity, attributing this enhancement to the incorporation of metallic nanoblocks into the device. It is imperative to underscore that the sensitivity of the device can be further augmented by refining the cavity shape to optimize SPP wave overlap with the ambient medium. Figure 8d,e showcase the normalized distribution of the E-field and H-field at the resonance wavelength, respectively. A closer examination of the magnified image of Fig. 8d reveals an intensified E-field confinement along the boundaries of the metallic nanoblocks, resulting in an enriched light-matter interaction. This spatially refined interaction contributes significantly to the overall sensitivity improvement of the device.

## Concluding remarks

Herein, we conducted a systematic numerical analysis of a cutting-edge plasmonic sensor designed for temperature sensing applications, employing a metal–insulator–metal (MIM) waveguide. The sensor’s design features a circular cavity that is filled with a thermo-optic material, specifically polydimethylsiloxane (PDMS). This cavity is side coupled to a MIM bus waveguide resulting in a ring resonator configuration.The transmission of light into and out of this nanoscale waveguide is efficiently managed through silicon-based orthogonal couplers. These couplers play a fundamental role in seamlessly transforming dielectric modes into plasmonic modes and vice versa. Such transformation capabilities enhance the sensor's performance and ensure optimal functionality. The proposed sensor designoffers a sensitivity of − 0.44 nm/°C over a temperature range spanning from 20 to 80 °C which can be further amplified to − 0.63 nm/°C by incorporating a periodic array of metallic nanodots in the center of the cavity. This characteristic makes it an excellent choice for precision temperature measurements. The outcomes of this study represent a significant step towards the growth and deployment of plasmonic sensing devices employing MIM waveguides, with the potential for a wide array of applications beyond temperature sensing.

## Data Availability

The authors declare that all data supporting the findings of this study are available from the corresponding author upon reasonable request.
